# Dysentery as It Prevailed in Meade County, Ky., in 1854

**Published:** 1855-03

**Authors:** H. K. Pusey

**Affiliations:** Garnettsville, Ky.


### Art. II.—DYSENTERY AS IT PREVAILED IN MEADE COUNTY, KENTUCKY, IN 1854.

BY H. K. PUSEY, M. D., OF GARNETTSVILLE, KY.

Having, during the present season, passed through a violent and protracted epidemic dysentery, I purpose to give a synopsis of my practice and observations in the disease. That the subject is one calling for renewed investigation no practising physician can doubt. Dysentery continues to be a most intractable and fatal disease. It is one of very frequent occurrence and of very general prevalence. For a number of years past, it has prevailed extensively as an epidemic in our country. Medical treatment has heen variant, contradictory, and often ineffectual. The profession is far from being agreed on the best method of combatting the disease. Under these circumstances any new facts that can be added to the experience of the profession will not be unacceptable.

In this region of country, up to the first of July, we had a great deal of rain, with luxuriant vegetation, but the general health continued good until about this time, when dysentery made its appearance, at first in a mild form. The disease has been limited to a district of country about six miles in extent, lying on the northeast side of Ott r creek, extending from the north of that stream about five miles up the Ohio river, and thence on a line parallel with the creek, about six miles back into the country, including the villages of Garnettsville and Grahamton, containing about four hundred inhabitants each. Outside of this district, though there have been cases of the disease, I have not heard of one of a malignant chararter. This fact indicates some local cause, but I have endeavored in vain to detect it. The country on the south of Otter creek is elevated, uneven and barren, with basins which, in wet weather, are filled with water, forming ponds, occasionally, of considerable size. These become the receptacles of the washings of the country, and their bottoms covered with decaying vegetable matter are exposed to the sun during the dry months of summer and early autumn. To these sources our fevers, which appear to be on the increase, have generally been ascribed. But such is not the character of the surface where dysentery prevailed so fatally. The country is high, broken, and free from stagnant water. None of the generally accredited causes of malarious disease exist there.

The number of cases under my care was one hundred and twelve, about one-third of which were obstinate and malignant, and occurred indiscriminately among subjects of both sexes, and all ages. The first symptoms were lassitude, pains in the head, bowels and limbs, constipation or diarrhea, coated tongue, heat, thirst, rigors; which continued usually for about twenty-four hours before tenesmus and bloody stools supervened. Most of the cases seemed to be a complication of remittent fever, but the febrile symptoms did not appear materially to affect the progress of the dysentery, which continued unchecked after the subsidence of the fever.

The treatment with which I was best pleased consisted of an emetico-cathartic, of ipecacuan and calomel, followed by opium and quinine. The relief from the operation of the first was generally decided, large quantities of bile being discharged from the stomach and bowels. If the bowels were not affected by the calomel, castor oil, or a saline cathartic was given until free discharges took place. In a few favorable cases the effect of this treatment was to arrest the complaint at an early stage, but for the most part it was merely palliative. As a general rule, the disease ran on for a period varying from six to twenty days. Opium was given to the extent of allaying pain and tenesmus, and with it I generally gave every day or two a blue pill, or a little hydrag. cum creta, followed by oil or rhubarb to empty the bowels. I treated satisfactorily a number of cases simply with syrup of rhubarb and cinnamon, in small, repeated doses. After the use of these articles for a short time, patients often complained of griping in the small bowels, but, at the same time, expressed themselves as feeling relieved from the distressing tormina. I employed morphia dissolved in warm water as an enema, and found the bowel much more tolerant of this mixture than the usual starch and laudanum injection.

Of the forty cases that occurred before the first of July, about six, after progressing from ten to eighteen days without any untoward symptom, assumed suddenly a typhoid type; with disorder of the stomach and upper bowels, red, smooth, dry, tongue, vomiting, precordial distress, tympanitis, but not much tenderness to the touch, pulse quick, feeble, extremities cold, wandering delirium, involuntary discharges from the bowels. The treatment pursued in these cases consisted in the free use of morphine, or Dover's powder, with stimulants, mucilaginous drinks, and nourishing diet. Anodyne injections, and anodyne poultices to the abdomen were also used, with dry cups, and in one or two cases blisters; but the latter did not appear to exert any beneficial influence. In the latter stage, nitrate of silver, given in doses of half a grain every four hours, sometimes appeared to act favorably. I derived no advantage from the use of the ordinary astringents, as tannin, acet. lead, &c.

Four of these cases terminated in recovery, and two ended unfavorably. Of the favorable cases, one recovered on the 20th day after the supervention of the typhoid symptoms, and the 32d day of the disease; one on the 36th day after the typhoid appearances, and the 47th day of the disease; and two on the 15th day after the typhoid complication, and the 27th day of the disease. Of the fatal cases, one died on the 7th day of the complication, and on the 30th day of the disease, with symptoms of pneumonia; and the other died from hemorrhage of the bowels on the 40th day of the disease, and the 24th from the access of typhoid symptoms.

During the last week in July, simultaneously with this unfavorable change in the type of the disease, there was a marked increase in the number of the cases, and from this time to the last of September, the disorder prevailed without the stages which characterized it in the first instance. About the same time remittent fever became prevalent, in some instances assuming a malignant or congestive form, but more generally taking on a continued or typhoid type. Dysentery from this time until it ceased to prevail was more unmanageable. The mode of attack in one class of cases was by diarrhœa, a brownish, watery discharge from the bowels, unattended by pain or uneasiness. In these cases the tongue was red, smooth and clean; pulse from 90 to 120, and rising in the progress of the disease, in some instances, to 130 or 140. These symptoms continued from two to four days before dysentery, or the bloody discharges appeared. This variety of the disease ran its course in from twenty to thirty-five days, and in one fourth of the cases proved fatal, by sudden profuse hemorrhages from the bowels, or general exhaustion. In one case, that of a young lady, on the 23d day of the disease, symptoms of perforation of the bowels occurred after convalescence had seemed to have commenced. The bloody stools generally ceased in from sixteen to twenty days, but no amendment followed their subsidence in the worst cases.

In this form of the complaint no active treatment was admissible. Nourishing diet, with brandy and stimulants, was the main reliance, with which sponging the surface was advantageously combined—opium was administered freely, so as to keep the patient slightly narcotized. Occasionally when the surface was hot, Dover's powder was given. Mercury was generally given every night, in the form of hydrag. cum creta or blue pill, and followed by syrup of rhubarb in the morning, when necessary. Sulphuric acid was used with benefit, as also tinct. mur. iron. In a majority of cases nitrate of silver was employed, after the febrile symptoms began to subside.

Another form in which dysentery presented itself was as a complication of congestive or malignant intermittent and remittent fever, and here the distinctive symptoms of the disease were less clear than in the typhoid variety, for the only evidence of dysentery, was the occasional discharge of bloody serum from the bowels, unattended with pain, or suffering of any kind. But the fact of these cases having occurred under the same circumstances and in the same families, with dysentery, justifies the conclusion that the hemorrhagic tendency, at least, was the result of a dysenteric impress upon the system. During the same time, similar cases of fever without the bloody stools were not uncommon. They however yielded more readily, to the influence of remedies; whilst the former, if the congestive symptoms were relieved, was exceedingly prone to pass into a typhoid state, and present appearances similar to those already described. The attack was sometimes sudden, but more commonly gradual. The patient complained of sluggishness and indisposition for a day or two. The extremities became cool and clammy, pulse feeble and frequent for twelve to twenty-four hours before the decided congestion and collapse came on, which was generally in the morning, and was attended by sinking of all the vital powers. The pulse became extremely feeble and irregular; extremities cold and clammy; the entire surface was pale and bathed in a cold sweat; the countenance presented an anxious, sunken and contracted appearance; voice hoarse and husky. The sensibilities were obtunded, the patient manifested but few wants, and complained of little or no pain or tenderness on pressure. The abdomen was hard and sunken—of the tongue there was no constant appearance; sometimes it was round, pointed and moist, of a bluish cast; sometimes natural, and in other cases dry and heavily coated. The discharges were composed entirely of serum colored with blood, containing little or no fecal matter or mucus, and occurred from six to eight times in twenty-four hours.

The duration of this form of the disease varied from 48 hours to an indefinite period,—that is when it ran into the typhoid variety, —the congestive symptoms however, rarely lasted longer than 48 to 72 hours, when reaction began to take place; except in three cases which died before the first named period—reaction was rarely completed and equalized in less than three or four days—during which time the brain, in one instance, the lungs in another, and the stomach in a third case, was involved in deep and threatening congestion, and indeed, the latter case subsequently proved fatal by gastritis.

The treatment relied upon in this variety of the disease was quinine in liberal quantities, and diffusible stimulants, sinapisms, stimulating frictions &c. Quinine in 10 gr. doses every three hours, with the above adjuvants in some instances relieved the patient after this formidable train of symptoms had set in. But the general and obvious tendency of the disease was to destroy life and what could be affected by remedies must be done principally in the forming stage, for after the congestion of the stomach and bowels took place also involving the brain, the nervous system did not seem to respond to, or recognize the presence of stimulants either internal or external.

In death from both varieties of flux, especially the congestive form, where reaction was not completed and death took place in a few days, the most extensive disorganization was observed, so much so as to render the sick room intolerably offensive even before death. The number of cases of both varieties was thirty-eight; 26 of the typhoid, and 8 deaths; and 12 of the congestive form, and 4 deaths.

*November*, 1854.

